# Basic Structures of Gut Bacterial Communities in Eusocial Insects

**DOI:** 10.3390/insects14050444

**Published:** 2023-05-08

**Authors:** Shota Suenami, Akiko Koto, Ryo Miyazaki

**Affiliations:** 1 Bioproduction Research Institute, National Institute of Advanced Industrial Science and Technology (AIST), Tsukuba 305-8566, Japan; 2 Computational Bio Big Data Open Innovation Laboratory (CBBD-OIL), AIST, Tokyo 169-8555, Japan; 3 Faculty of Life and Environmental Sciences, University of Tsukuba, Tsukuba 305-8572, Japan

**Keywords:** social insects, gut microbiota, bacterial community structure

## Abstract

**Simple Summary:**

It is increasingly recognized that gut microbiota plays crucial roles in host health and function. Various ecological and physiological factors influence the structure of the gut microbial community, resulting in, for example, the formation of enterotypes or the development of inflammatory disease by dysbiosis in humans. Social insects, such as bees, ants, and termites, are known to harbor unique but stable gut microbiota among individuals, which can be a good model to understand how gut microbial communities are shaped and stably maintained in host populations. This review summarizes current knowledge regarding structures of gut microbiota in social insects. Microbes colonizing those insect guts and differentially abundant among host castes are mainly featured.

**Abstract:**

Gut bacterial communities assist host animals with numerous functions such as food digestion, nutritional provision, or immunity. Some social mammals and insects are unique in that their gut microbial communities are stable among individuals. In this review, we focus on the gut bacterial communities of eusocial insects, including bees, ants, and termites, to provide an overview of their community structures and to gain insights into any general aspects of their structural basis. *Pseudomonadota* and *Bacillota* are prevalent bacterial phyla commonly detected in those three insect groups, but their compositions are distinct at lower taxonomic levels. Eusocial insects harbor unique gut bacterial communities that are shared within host species, while their stability varies depending on host physiology and ecology. Species with narrow dietary habits, such as eusocial bees, harbor highly stable and intraspecific microbial communities, while generalists, such as most ant species, exhibit relatively diverse community structures. Caste differences could influence the relative abundance of community members without significantly altering the taxonomic composition.

## 1. Introduction

All eukaryotes are in association with microorganisms. In animals, the gut is especially important for microbes, as it is a nutrient-rich environment. Many gut microbes are symbionts of their hosts and provide numerous benefits, such as improved metabolism and immunity [1]. However, host animals can also accidentally acquire pathogens or opportunistic microbes from the environment. To maximize the benefits of gut symbionts, it is therefore important for hosts to properly shape and stably maintain their gut microbiota. Social animals have the unique characteristics that individuals in a population have similarly structured gut microbiota, implicating that social interactions between members help to share microbes across the population [2,3]. This stability of gut microbiota among individuals makes social animals a useful model to study basic principles that control the structure, function, and evolution of gut microbial communities. However, because a large number of bacterial species compose mammalian gut microbiota (e.g., approximately 300 operational taxonomic units (OTUs) per individual mouse [4]), the biological interactions between microbes and hosts are highly complex, making it difficult to elucidate the fundamental mechanisms that shape the gut microbiota.

In contrast to mammals, insects generally harbor simpler gut microbiota [5]. Several gut microbes are known to be beneficial to the host insects [6,7,8], although they can be lost during development. Insects inevitably undergo molting (all species) and pupation (only holometabolous species), during which gut microbes are lost with the shedding of the gut epithelium [5,9,10]. Reinoculation is thus required to gain and maintain beneficial gut microbes, but the basic lifestyle of insects often poses challenges for the transfer of microorganisms between generations: most insects are classified as solitary, with females abandoning their eggs after laying, and thus opportunities to transfer gut microbes between conspecifics are limited.

To avoid those risks, several insects have evolved unique systems for the stable transfer of their symbiont microorganisms. Many endosymbionts, obligate symbiotic bacteria associated with specific insects, undergo maternal germ-line transmission to offspring [11]. Some obligate symbionts are known to be transmitted vertically via specific capsules deposited by adult females upon oviposition and then taken in by newborn nymphs [12]. Another example, but probably the most successful system, is that of social insects, including termites, ants, and some wasps and bees, which have evolved gregarious or social lifestyles. They share the living space and show social interactions such as oral/proctodeal trophallaxis or coprophagy, which allow indirect or direct transmission and maintenance of gut symbionts within their populations [13,14,15]. Consistent associations between gut microbes and social insects lead to the hypothesis that they have coevolved [16,17,18].

Gut microbiotas of social insects have been reviewed in several works of literature by insect groups, such as social bees or termites [19,20,21]. In the present review, we overview the gut microbiota of a wide range of eusocial insects together and summarize their structures at a glance to gain insight into any general/common aspects of their structures. Our focus is on intestinal bacterial communities rather than specific endosymbionts whose characteristics have been reviewed elsewhere [22,23,24]. Because of a recent increase in 16S rRNA amplicon sequencing studies, we discuss their communities mainly at the 16 rRNA phylotype level.

## 2. General Ecology of Social Insects

The type of insect sociality is categorized by the degree of cooperation [25]. The highest level of sociality is found in eusocial insects such as ants, termites, and some bees and wasps, which are characterized by three traits of sociality: 1. Cooperative brood care, 2. reproductive division of labor, and 3. overlapping generations. A general lifestyle of eusocial insects is explained here with ants as an example. Ants form a colony consisting of one or more reproductive queens, many non-reproductive female workers, and a small number of males, which are dedicated to reproduction [26]. Workers have a marked division of labor, with individuals performing specific tasks within a colony, such as foraging, nest construction, and nursing. The triggers for individual task preference vary among social insects. In ants, it is generally correlated with age: younger workers stay in the nest for brood care, and older workers leave the nest to forage [26]. On the other hand, morphology influences task allocation in bumble bees and stingless bees [27,28]. In addition to the age-related task polyethism, individual workers of ants also change their roles flexibly according to the needs of their colony [29,30].

Social insects are also categorized according to a wide range of dietary ecology, from herbivorous to omnivorous and carnivorous. Herbivorous insects utilize plant sources such as nectar, pollen, wood, and grass. Most eusocial bees and termites belong to this group. Carnivorous insects, such as predatory army ants, use other animals as their food. Omnivorous insects are versatile as they can feed on both plant and animal sources. Some ants and wasps belong to this category. This diet ecology could, to some extent, be related to gut bacterial community structures, as we discuss in the following sections.

## 3. Eusocial Bee Gut Microbiota

Eusocial bees belong to the order Hymenoptera [31]. Eusocial bee species (e.g., *Apis* honey bees, *Bombus* bumble bees, and *Tetragonula* stingless bees) have similar but not identical social systems to each other. For example, honey bees, like ants, show age-dependent polyethism, whereas bumble bees show it related to morphology rather than age (see Section 2). Task allocation in stingless bees is influenced by both age and morphology [28,32]. Most eusocial bees are herbivorous, collecting nectar for carbohydrates and pollen for proteins, lipids, vitamins, and other macronutrients [33]. The digestive tract of eusocial bees consists of the crop for the temporary storage of food, the midgut for digestion and absorption of food, and the hindgut, which consists of the ileum and rectum, for removal of water and nutrients and preparation for excretion [5,34]. Honey bees are insects whose gut microbiota have been extensively studied [19].

### 3.1. Apis mellifera and Other Honey Bees

**Figure 1:**
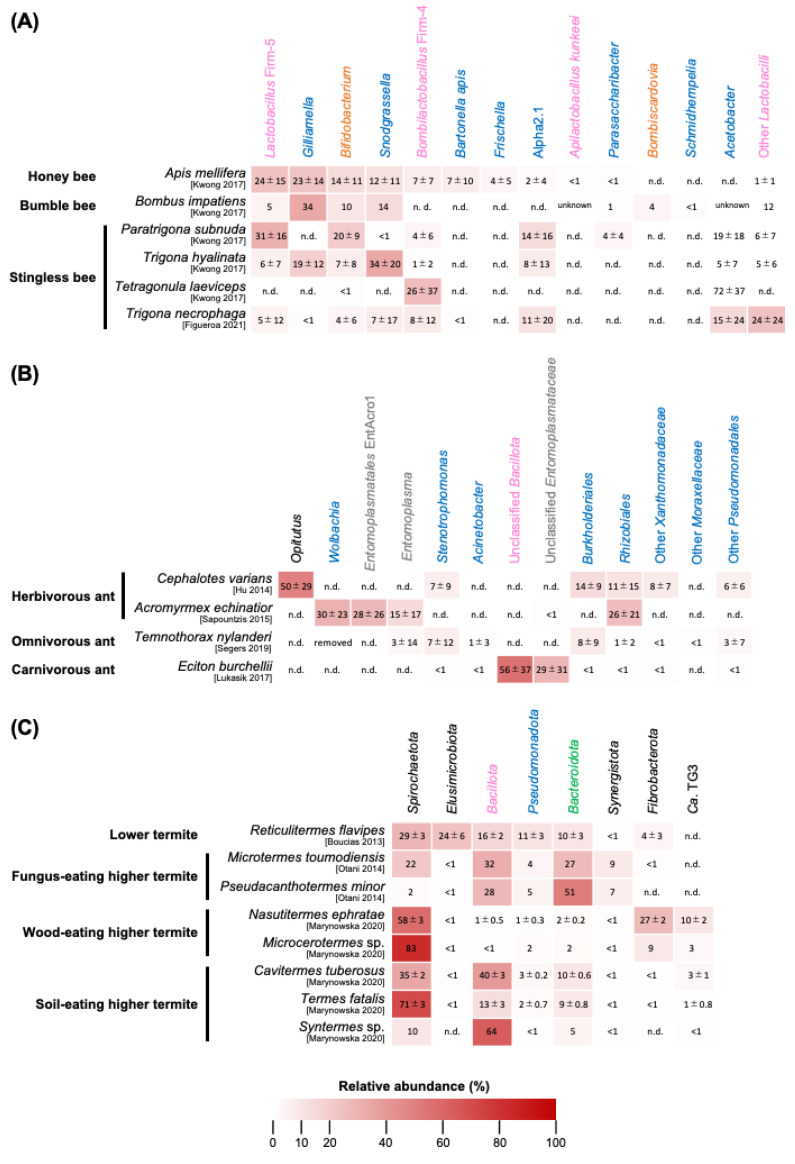
Structural profiles of gut microbial communities in eusocial insects. Heat map illustrates average relative abundance with standard deviations of bacterial taxa in representative eusocial bees (**A**), ants (**B**), and termites (**C**). Only phylotypes discussed in the text are presented with different colors at the phylum level: blue, *Pseudomonoadota*; magenta, *Bacillota*; orange, *Actinomycetota*; green, *Bacteroidota*; grey, *Mycoplasmatota*; and black, other phyla. References are indicated in square brackets [38,48,49,50,51,52,53,54,55]. Average and standard deviation are calculated assuming a Gaussian distribution of the population. Metadata of each sample are summarized in Supplemental Table S1. n.d., not detected.

The genus *Apis* consists of approximately 10 species [35,36], of which the European honey bee, *A. mellifera*, is the most studied species for its gut microbiota. *A. mellifera* establishes a larger colony than other *Apis* species, with an average of 100,000 individuals [37]. Honey bee colonies reproduce by swarming, through which a single colony splits into two or more colonies with newly mated queens. In the whole gut of female workers, more than 80% of the bacterial community is represented by five dominant (or “core”) phylotypes (Figure 1A) [19]: *Snodgrassella* (*Betaproteobacteria*), *Gilliamella* (*Gammaproteobacteria*), *Bombilactobacillus* Firm-4 and *Lactobacillus* Firm-5 (*Bacilli*), and *Bifidobacterium* (*Actinomycetes*), comprising approximately 12, 23, 7, 24, and 14% of the community, respectively [38]. *Frischella* (*Gammaproteobacteria*) and *Bartonella* (*Alphaproteobacteria*) are less abundant than the five core phylotypes, accounting for 4 and 7% of the bacterial biomass, respectively [38]. Interestingly, these seven phylotypes are bee-specific lineages that are restricted to bees and their nest environment [19,39]. They are known to be socially transmitted between *A. mellifera* individuals [15], but have also been detected in other *Apis* species with different abundance and/or prevalence among host species [38]. A typical example is that prevalence and relative abundance of *Snodgrassella* are strikingly low in *Apis dorsata* workers (prevalence, 0–20%; relative abundance, 1% or less). Although the mechanism underlying gut microbiota diversification among *Apis* species is unknown, their ecology, such as population size per colony and nest openness, could be involved in the difference in gut microbiome structure [36,37].

Functions of some gut bacteria relevant to their presence in *A. mellifera* have been experimentally demonstrated. *Gilliamella* produces pectin-degrading enzymes for pollen breakdown and utilizes monosaccharides derived from natural nectar, such as mannose and xylose, which can be toxic sugars to hosts [33,40]. Pollen processing is also supported by other community members [8]. In addition to those contributions to host nutrition, gut bacteria stimulate the production of host hormones known to impact bee development and growth [8,41] or activate the host immune system [42,43].

**Figure 2:**
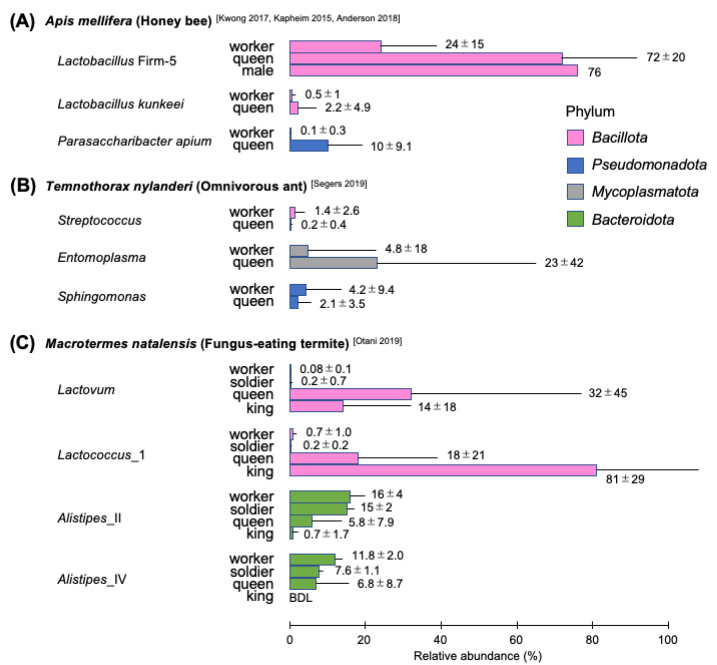
Differentially abundant microbes among host castes. Bar plots show average relative abundance with standard deviations of representative bacterial lineages, which exhibit more than 1% abundance in either caste and more than 2-fold change in the abundance between castes. Gut samples from natural colonies of *Apis mellifera* (**A**), *Temnothorax nylanderi* (**B**), and *Macrotermes natalensis* (**C**) are used. Note that bacterial lineages are described with different taxonomic levels, following references that are indicated in square brackets [38,44,47,55,59]. Average and standard deviations are calculated assuming a Gaussian distribution of the population. Metadata of each sample are summarized in Supplemental Table S2. BDL, below the detection limit.

As the age-dependent polyethism in honey bees involves changes in the physiology and working environment of workers, one might assume that it also influences the structure of the gut microbiota. However, the community structure is similar among *A. mellifera* workers regardless of age and task in the natural colony [44,45]. When ‘single-cohort’ colonies were experimentally established to investigate the influence of task on gut microbiota independent of age, a significant difference in the relative abundance of some phylotypes was detected between nurses and foragers: *B*. Firm-4, *L.* Firm-5, and *Bifidobacterium* are more abundant in nurses than in foragers, while an opportunistic bacterium *Apilactobacillus kunkeei* becomes more abundant in foragers than in nurses [46]. Therefore, different tasks could influence the gut microbiota of workers, although age-related physiological changes would mask the effect. In addition, a few phylotypes, *L*. Firm-5, *A. kunkeei*, and *Parasaccharibacter apium*, are known to be more abundant in reproductive castes (i.e., queens and males) than in workers (Figure 2A) [44,47]. Although the mechanisms underlying the difference have not been well understood, their physiology related to reproduction and/or the diet provided by workers may affect it.

To what extent gut microbiota is stable against environmental changes is of interest in understanding community robustness. While geography does not have a large effect, seasonality influences the abundance of core phylotypes in *A. mellifera* workers [19,56,57,58]. Pollen consumption is known to increase the absolute abundance of most phylotypes in laboratory-reared workers [58]. Thus, changes in diet or climate alter the abundance of core phylotypes but not their composition.

Overall, the gut microbiota of *A. mellifera* and other *Apis* species consists mainly of the five bee-specific core phylotypes, which are socially transmitted. The structure is generally consistent among individuals, but the relative abundance of core phylotypes differs between reproductive and non-reproductive castes. Seasonality and diet, but not geography, also influence that abundance.

### 3.2. Bumble Bees

There are more than 250 species of bumble bees over the world [60]. Bumble bees establish colonies that are smaller than those of *A. mellifera* and house a single queen, female workers, and males [60,61]. Unlike honey bees, colony foundation is carried out by the queen alone, suggesting that the queen is the source of gut microbiota for offspring in the same colony [62]. Bumble bee workers have a similar gut microbiota to that of honey bees in terms of core phylotypes (Figure 1A). In seven bumble bee species investigated so far, two core phylotypes, *Snodgrassella* and *Gilliamella*, are detected with a relatively high prevalence of >60% and >40%, respectively, while the prevalence of Gram-positive phylotypes (*Lactobacillus* and *Bifidobacterium*) is variable across the host species [38]. *B*. Firm-4 is noticeable as it is not detected in most of the seven bumble bee species. The gut microbiota of bumble bees is generally dominated by *Pseudomonadota* (formerly “*Proteobacteria*”), and relative abundances of *Snodgrassella* and *Gilliamella* are 14–55% and 15–49%, respectively [38]. Some bumble bee species have two *Bombus*-specific phylotypes, *Bombiscardovia* (*Actinomycetes*) and *Schmidhempelia* (*Gammaproteobacteria*) (Figure 1A) [38,63,64]. The functions of gut microbes in bumble bees are not well understood, but there are a few reports investigating their involvement in pathogen inhibition, learning, and memory [65,66,67]. Interestingly, some bumble bee species are known to have two enterotypes in nature, in contrast to *A. mellifera*, which has a single enterotype [68]. One of the enterotypes is enriched with the common phylotypes described above, while the other is characterized by *Serratia* and *Hafnia* (*Gammaproteobacteria*), which include insect pathogens [68]. The lack of the common phylotypes in the latter enterotype might be related to the hibernation of queens or the invasion of foragers by environmental bacteria [68], although it is unknown whether the enterotype is disadvantageous to bumble bees.

Differences in gut microbiota among castes of bumble bees have not been well investigated. A preliminary report shows that a *Bombus pascuorum* queen, foragers, and inhive workers share a similar microbiota [69]. This is consistent with the above hypothesis that gut microbiota is transferred from a colony-founding queen to her offspring [62], although further studies with different samples and colonies are needed. In queens of *B. lantschouensis* and *B. terrestris*, the abundance of opportunistic bacteria varies depending on their life stages, such as aging and hibernation [70,71]. Such changes in the queen’s gut microbiota could be crucial for colony fitness, as her microbiota could be directly transmitted to her offspring.

In short, bumble bee workers have similar core phylotypes to those of honey bees, with the exception of *B*. Firm-4, which is absent in most of the bumble bee species. A unique feature of bumble bees is that some species have two enterotypes, one of which contains possible insect pathogens. The gut microbial structure is largely similar among castes, although the queen’s gut microbiota is known to change during her life cycle.

### 3.3. Stingless Bees

This bee lineage consists of 60 genera with 600 species, which is a larger group than other eusocial bees [72]. The colony size of stingless bees varies from a few hundred to ten thousand workers, depending on the species [73]. Stingless bees generally have an age-related task allocation similar to honey bees, while a few species exhibit a morphology-based division of labor [32,74]. The gut microbiotas of stingless bees are more variable among species, compared to those of other eusocial bees. A previous study has reported gut bacterial community profiles of 13 Meliponini species, where none of the five core phylotypes are consistently detected [38]. Of the 13 species, 5 harbor only one or two core phylotypes; for example, *Tetragonula laeviceps* has only *Bombilactobacillus* Firm-4 and *Bifidobacterium* (Figure 1A). Most of the Meliponini species, on the other hand, harbor an *Acetobacter*-like phylotype that is seldom detected in other eusocial bees. These differences between stingless bees and other eusocial bees seem to be due to the loss and gain of these phylotypes during host bee evolution [38], which is further discussed in the recent study using neotropical stingless bees [75].

It has also been proposed that stingless bees have undergone ecological shifts which have released hosts from reliance on the five core phylotypes or the acquisition of new microbes performing functions that have been carried out by the previous phylotypes [76]. Intriguingly, in contrast to honey bees and bumble bees, which are all pollinivores, stingless bees contain necrophagous species that consume vertebrate carcasses [77]. An obligate necrophage, *Trigona necrophaga,* harbors some of the core phylotypes but also more abundant *Acetobacter* and lactic acid bacteria (Figure 1A) [49]. This could be a typical example that the extreme diet switch from pollen to carrion was likely facilitated by or resulted in the novel composition of the stingless bee microbiota.

While the consistency of the gut microbiota of stingless bees has not been fully investigated experimentally, one study has reported that colony relocation significantly changed the bacterial community in *T. carbonaria* [78]. Although further surveys with other stingless bee species are needed, it suggests that their gut microbial communities might be less stable than those of honey bees or bumble bees, and could be easily altered by environmental disturbance.

### 3.4. Summary of Bee Gut Microbiota

Eusocial bee gut microbiotas generally exhibit a simple taxonomic composition. Honey bees have five core phylotypes (*Snodgrassella*, *Gilliamella*, *Bifidobacterium, Bombilactobacillus* Firm-4, and *Lactobacillus* Firm-5), most of which are shared with bumble bees and stingless bees, suggesting that a common ancestor of those eusocial bees had harbored them. This hypothesis is further supported by the fact that the host phylogeny is largely congruent with trees based on phylogenetic marker genes of the core phylotypes [18,38]. In addition, *Apis*, *Bombus*, and stingless bees have also acquired their own lineage-specific bacteria. These indicate that the gut microbiotas of eusocial bees have coevolved with their host species. On the other hand, the relative abundance of gut microbes could vary between castes (e.g., queens and workers of *A. mellifera*) (Figure 2A), between different life stages (e.g., queens of bumble bees), or under external conditions (e.g., seasonality and environmental changes associated with colony relocation). These suggest that some physiological or ecological contexts of individual bees could also influence the gut bacterial community structure, although it is highly consistent among individuals within the same caste. As a number of studies have focused only on *A. mellifera* and a few bumble bee species, further investigations on other social bees would provide new insights into physiological and ecological mechanisms shaping their gut microbiota.

## 4. Ant Gut Microbiota

Ants belong to Formicidae in Hymenoptera and dominate the terrestrial ecosystems around the world. They have been diversified into over 13,000 species, all of which are eusocial [79]. The basic structure of the digestive tract is similar to that of eusocial bees, although some ants have modified structures, such as pouches where commensal bacteria densely colonize [80,81]. Ants have distinct gut microbiota, depending on their diet. Broad surveys of ant gut microbiota suggest a clear trend that specialists (e.g., herbivores) have increased bacterial density but reduced bacterial diversity in their guts. On the other hand, generalists (e.g., omnivores) tend to have a low abundance of gut bacteria [50,51,79,82].

### 4.1. Herbivorous Ants

Species belonging to the genus *Cephalotes*, especially *Cephalotes varians* turtle ants, have been well investigated for their gut microbiota. They are herbivores that favor plant exudates, honeydew, and pollen [83,84]. Phylotypes belonging to *Opitutales* (*Verrucomicrobiota,* formerly “*Verrucomicrobia*”), *Xanthomonadales*, *Burkholderiales*, *Rhizobiales*, and *Pseudomonadales* (*Pseudomonadota*) are commonly detected in the guts of *Cephalotes* ants [83,85]. *Opitutus,* belonging to the *Opitutales*, is predominant (on average 50% of relative abundance) in *C. varians*, followed by *Xanthomonadaceae* (15%), *Burkholderiales* (14%), *Rhizobiales* (11%), and *Pseudomonadales* (6%) (Figure 1B) [50]. These observations suggest adaptation and coevolution of these phylotypes to *Cephalotes*, which is further supported by phylogenetic analyses where these phylotypes form *Cephalotes*-specific clades [7,50]. Indeed, a previous study has experimentally demonstrated that those gut microbes help *C. varians* workers obtain amino acids from dietary urea [7]. *C. varians* workers from natural and laboratory-reared colonies exhibit a similar composition of gut microbiota, while pollen feeding significantly increases the relative abundance of *Rhizobiales* but decreases that of *Burkholderiales*, suggesting that the feeding condition could alter the gut microbiome structure [50].

Fungus-growing ants are a group of herbivorous species which cultivate fungi in the colony for food. While some of them are known to harbor bacteria on their body surface, possibly to protect themselves and their habitats from infectious diseases [86], studies on their gut microbiota have been limited to two genera of leafcutter ants, *Acromymex* and *Atta*, which are infected by the endosymbionts *Wolbachia* (*Rickettsiales*) and/or EntAcro1 (*Entomoplasmatales*). In *Acromymex echinatior*, *Acromymex octospinosus*, and *Acromymex volcanus*, OTUs belonging to *Wolbachia*, EntAcro1, *Entomoplasma* (another *Entomoplasmatales*), and *Rhizobiales* occupy >97% of their gut microbiome (Figure 1B) [54]. *Atta cephalotes* workers are also infected by EntAcro1, of which relative abundance varies from <0.07% to 99.9% among specimens [87]. At low levels of EntAcro1 (i.e., <0.07% relative abundance in *A. cephalotes* workers), the gut microbiota is occupied by commensal bacteria such as *Rhizobiales*, *Pseudomonas* and *Pelomonas* (*Pseudomonadota*), *Staphylococcus* and *Lactococcus* (*Bacillota*), *Chryseobacterium* (*Bacteroidota* formerly “*Bacteroidetes*”), and *Cyanobacteria* [87]. Although the cause of the difference among *A. cephalotes* workers is still unknown, it is possible that EntAcro1 and other commensal bacteria are in an antagonistic relationship.

### 4.2. Omnivorous Ants

Ants are unique insects compared to bees and termites in that they include not only herbivorous but also carnivorous and omnivorous species. Omnivorous ants, such as *Azteca* and *Crematogaster*, are known to have few or no gut bacteria [79,82]. While the reasons for the low bacterial density are still under debate, the literature suggests that not all species require a functional gut microbial community [88,89]. On the other hand, dense gut microbiotas are found in some omnivores, such as *Temnothorax nylanderi*, of which workers harbor a number of OTUs (646 OTUs excluding *Wolbachia*) in the abdominal microbiota (Figure 1B) [55]. The structure of the gut microbiota is known to change with the seasons or laboratory-rearing conditions, although *Stenotrophomonas*, *Acinetobacter*, and other genera belonging to the *Xanthomonadaceae* or *Moraxellaceae* families are consistently detected irrespective of those conditions, suggesting that these microbes are stably maintained in the workers. A few bacterial lineages appear to be differentially abundant in workers and queens of *T. nylanderi* (Figure 2B), although further sampling and statistical analyses are required.

*Camponotus* carpenter ants are also known to be omnivorous based on their dietary habits, although they are classified as herbivores based on trophic measurements [79,85]. The abdominal microbiota of *Camponotus chromaiodes* is dominated by two *Acetobacteraceae* OTUs (with approximately 80% of reads after removing massive amounts of *Candidatus* Blochmannia and *Wolbachia* endosymbionts reads), which are detected in distinct colonies, castes, and other *Camponotus* species such as *Camponotus castaneus* [90]. The two OTUs form a deeply divergent, monophyletic clade with other ant-associated *Acetobacteraceae* OTUs [90], although they are reduced in laboratory-reared *C. japonicus*, suggesting that the OTUs are not always required in the host [91]. *C. japonicus* queens have a more consistent community than workers and males. The community structure does not differ significantly among colonies [91].

In the omnivorous bullet ant, *Paraponera clavata*, collected in four Central or South American countries, two *Tumebacillus* (*Bacillota*) OTUs are prevalently detected [92]. When the ant is maintained in the laboratory with sterilized sucrose and water, one *Granulobacter* (*Pseudomonadota*) OTU, four *Asaia* (*Acetobacteraceae*) OTUs, and one *Frateuria* (*Xanthomonadaceae*) OTU, all of which are minor in the field samples, are detected in more than 50% of individuals, while the two prevalent OTUs are still present. The shift towards the *Acetobacteraceae*-biased community might reflect adaptation to a sugar-rich diet [92].

### 4.3. Carnivorous Ants

Although a number of ant genera are known to be specialists in animal prey, army ants are the best-studied group of carnivorous ants on their gut microbiota. Army ants are characterized by a strikingly simple and specialized microbiota in their gasters. In 40 army ant species (e.g., *Eciton burchellii*, *Labidus praedator*, and *Aenictus gracilis*) collected in the New World and Old World, 89% of specimens have three or fewer OTUs at >1% relative abundance [51]. Unclassified *Bacillota* and unclassified *Entomoplasmataceae* represent approximately 58% and 22% of total reads, respectively, regardless of host species and geography (Figure 1B). Based on phylogenetic analyses, these OTUs are hypothesized to have been already harbored by the common ancestor of New and Old World army ants, which diverged approximately 87 million years ago [51]. This suggests that army ants are highly invested in symbioses with the dominant bacteria, although their stability against environmental disturbances and functions of them need to be experimentally investigated. Interestingly, 16S rRNA amplification is not always successful with individuals from the same colony or across species in the same genus (e.g., the success rate was 90% for *Labidus praedator*, but only 15% for *Labidus coecus*) [51,82]. These suggest that not all individuals of army ants harbor gut bacteria, which is consistent with the cases of some omnivorous ants.

### 4.4. Summary of Ant Gut Microbiota

Ants with narrower dietary ecologies, i.e., herbivorous and carnivorous species, harbor simple gut microbiota dominated by a small number of phylotypes (Figure 1B). These dominant microbes are often shared among conspecifics in nature, while some environmental factors, such as food, alter the gut microbiome structure. It is thus implied that a more restricted dietary ecology is associated with the presence and maintenance of such unique microbes. On the other hand, omnivorous ants are known to have few or no gut microbes, but this is not always the case. Some species have substantial gut microbes which remain associated with the host. *Acetobacteraceae* OTUs are repeatedly detected in several omnivorous ants and form a monophyletic group within the ant-associated cluster, suggesting that a common function of these OTUs might be important in their ecology.

Although the influence of host-rearing conditions on ant gut microbiota is often studied, other ecological aspects, such as differences in gut microbiota between individual types (e.g., castes and sexes), would garner much interest but have not been examined in most ants. Extremely low or absent gut microbes in some omnivorous and carnivorous ants is another topic to understand the eco-evolutionary relationships between ants and microbes. Unlike eusocial bees, which also belong to the order Hymenoptera, the presence of endosymbionts in some species could be a unique feature of ants, although studies on interactions between such endosymbionts and luminal bacteria are limited.

## 5. Termite Gut Microbiota

Termites are a large group of insects in the order Isoptera with more than 3000 species [93]. Their social structure is very different from those of bees and ants. In a termite society, only the reproductive castes (kings and queens) are adults, while workers and soldiers are larvae of males and females. Termite workers and soldiers can develop into reproductives, unlike bees or ants, whose workers cannot replace their queens. Termites are traditionally categorized as higher or lower termites: higher termites represent species in the family Termitidae, which includes approximately 80% of all termite species, while lower termites belong to other families [93,94]. This categorization is also related to the presence of symbiotic gut flagellates, which are protists that help digest lignocellulose in the gut. Lower termites have flagellates and feed exclusively on wood and/or grass, while higher termites, which do not have flagellates, show a wider range of feeding habits, e.g., on fungi, humus, soil, or wood [20,93,95]. The evolutionary changes in feeding habits between lower and higher termites could explain the loss of flagellates and their replacement by a greater diversity of prokaryotes in higher termites.

Termites are important degraders of lignocellulose in terrestrial ecosystems [20]. Lignocellulose digestion is a complex process involving the degradation of recalcitrant molecules, for which higher termites compartmentalized their hindguts into P1 (ileum), P2 (enteric valve), P3–P4 (colon), and P5 (rectum) [20,96,97]. The structure and function of these compartments differ among termite species in accordance with their feeding ecology. Lower termites and fungus-eating termites do not have a P1, while the P1 of the soil-feeding higher termites exhibits pronounced alkalinity to process humic acids. The compartments also differ in redox potential and partial pressures of oxygen and hydrogen [20].

Symbiosis with flagellates, feeding habitat, and physicochemical properties of the gut are associated with gut bacterial community structure. We discuss here some termite bacterial communities at higher taxonomic levels (e.g., phylum), as bacterial communities in termites are less characterized than in bees and ants.

### 5.1. Lower Termites

In the gut of lower termites, flagellates are generally associated with endosymbiotic and ectosymbiotic bacteria [20,98]. This corresponds to the frequent occurrence of specific bacterial taxa, such as *Treponema* (*Spirochaetota,* formerly “*Spirochaetes*”) and *Endomicrobia* (*Elusimicrobiota,* formerly “*Elusimicrobia*”). *Reticulitermes* are well-studied lower termites in terms of their gut microbiota. Soldiers and/or workers of *Reticulitermes speratus*, *Reticulitermes grassei*, and *Reticulitermes flavipes* have abundant *Spirochaetota*, *Elusimicrobiota*, *Bacillota*, *Bacteroidota*, and *Pseudomonadota*—in *R. flavipes* hindgut fluid, the relative abundance of these phyla are approximately 25, 20, 18, 14, and 11%, respectively (Figure 1C) [13,48,99]. *Spirochaetota*, especially *Treponema*, is the most dominant symbiont in these *Reticulitermes* species. The composition of the hindgut fluid bacterial community in *R. flavipes* is not significantly altered by dietary treatments, although a large proportion of the microbiota (40% of relative abundance) could not be annotated at taxonomic levels lower than phylum [48].

*Stolotermes ruficeps* is one of the most phylogenetically basal termites [100,101]. Nymphs (a temporary worker caste) collected from different *S. ruficeps* colonies have similar gut bacterial communities with each other: *Bacteroidota* and *Spirochaetota* are the most dominant phyla, followed by *Elusimicrobiota*, *Pseudomonadota*, and *Bacillota* (46, 31, 9, 9, and 3% of relative abundance, respectively) [101]. While the overall composition of the gut microbiota is consistent with those of other lower termites, a relatively high abundance of *Bacteroidota* is characteristic of *Stolotermes* (average relative abundance in other lower termites; *Bacteroidota*, 13%; *Spirochaetota* 49%; *Elusimicrobiota* 7%; *Pseudomonadota* 7%; and *Bacillota* 11%). This might be related to the feeding habits of *Stolotermes*, which favor decaying wood colonized by fungi [101,102].

### 5.2. Fungus-Eating Higher Termites

This termite group, consisting only of the subfamily Macrotermitinae, cultivates symbiotic fungi on the plant material, which is partially degraded by the fungi and provided to the termites [93,103]. Gut bacterial communities of nine fungus-growing termite species are highly dominated by *Bacteroidota* and *Bacillota*, followed by *Spirochaetota*, *Pseudomonadota*, and *Synergistota* formerly “*Synergistetes*” (average 32, 34, 9, 9, and 7% of relative abundance, respectively) (Figure 1C) [53]. In those phyla, the most abundantly and prevalently detected genera are *Alistipes* 1 (*Bacteroidota*, 10.9% of relative abundance), *Treponema* 1a (*Spirochaetota*, 5.6%), *Ruminococcaceae* Gut Cluster 1 (*Bacillota*, 4.3%), Ca. Tammella (*Synergistota*, 3.9%), and *Desulfovibrio* 3 (*Pseudomonadota*, 3.3%). Comparable results have been observed in several studies [95,102,104]. The dominance of *Bacteroidota* and *Bacillota* and the relatively high abundance of *Synergistota* in Macrotermitinae are similar characteristics to those of omnivorous cockroaches rather than in other termites, suggesting that the unique deeding habits rather than host phylogeny shape gut microbiota of these insects [53].

A number of OTUs are shared in all castes of *Odontotermes* spp. or *Macrotermes natalensis*, both belonging to the Macrotermitinae, but some OTUs are differentially abundant among them (Figure 2C) [59]. In particular, queens and kings of fungus-growing termite species are unique in that their microbiota are reduced in diversity and dominated by only 1–3 bacterial genera, resulting in significant differences in gut microbiome structures between reproductive and non-reproductive castes.

### 5.3. Wood/Soil-Eating Higher Termites

Gut bacterial communities of wood-feeding higher termites are dominated by *Spirochaetota* (average 64% of relative abundance) and two specific phyla, *Fibrobacterota,* formerly “*Fibrobacteres*”(17%) and candidate phylum TG3 (10%), but the abundances of *Bacillota* and *Bacteroidota*, which are widely distributed in higher termites, are much lower (Figure 1C) [52]. On the other hand, soil-feeding higher termites consistently harbor *Bacillota* (35%) and *Bacteroidota* (8%), as well as *Spirochaetota* (38%) as dominants (Figure 1C). Further classification using a curated reference database reveals that *Treponema* I, TG3 Termite Cluster III, and *Fibrobacterota* Termite Cluster I are frequently detected families in wood/grass-feeding termites, while two genera from *Lachnospiraceae* (Gut Cluster 13 and Ca. Arthromitus) are abundantly detected in soil-feeding termites [95,105]. Although little is known about the relevant functions of these microbes in host colonization, it has been experimentally demonstrated that the degradation of xylan, a major component of hemicellulose, is performed by the dominant *Sprirochaetota* in a wood-feeding higher termite *Nasutitermes* [106].

The difference in community structures is based on distinct bacterial communities in the gut compartments of these termites. P1, which shows high alkalinity [20], harbors abundant *Bacillota* regardless of host diets (40–49 and 63–75% of total reads obtained from P1 of wood/grass feeders and humus/soil feeders, respectively) [96]. On the other hand, bacterial communities in P3 contain more *Spirochaetota* in wood/grass feeders than in humus/soil feeders (55–76 and 4–19% of total reads from P3, respectively). Wood feeders also harbor relatively higher amounts of *Fibrobacterota* and TG3 (3–7, and 5–18% of reads, respectively) than grass/humus/soil feeders (<1% for both phyla), while humus/soil feeders have more abundant *Bacillota* than wood/grass feeders (at 50–61 and 3–21% relative abundance, respectively). In P4, both humus and soil feeders have a high abundance of *Bacillota* (38–57% of reads), but humus feeders have a higher abundance of *Bacteroidota* (25–34%) compared to soil feeders (5–13%). Wood/grass feeders exhibit variable bacterial communities in P4 without any obvious trends.

### 5.4. Conclusions of Termite Gut Microbiota

Overall, lower and higher termites share similar gut microbial communities at the phylum level, which are dominated by *Spirochaetota*, *Bacteroidota*, and *Bacillota*. On the other hand, *Elusimicrobiota* is a specific phylum for lower termites, while *Fibrobacterota* and TG3 are detected mainly in wood-eating higher termites (Figure 1C). Compared to bees and ants, it seems that more host species have been investigated for termite gut microbiota. This allows us to infer the evolutionary trajectory of the gut microbiota across termite species. Although the cladogram of the gut bacterial community largely discriminates between lower and higher termite groups, the internal topology of the cladogram often does not match the host phylogeny [102]. This suggests that termite gut microbiota has drastically changed through lower/higher termite transition but consists of a mixture of both bacterial lineages acquired through vertical and horizontal transmissions [107], and that those microbes horizontally transferred among colonies or from the environments could diversify termite communities.

Differences in gut microbiotas among termite castes, colonies, or geographical areas have been examined in limited studies (Figure 2C) [13,48,59,101]. There are still many reports that pooled worker termites as a sample, but knowledge of the gut microbiome structure and dynamics at the level of individual termites should provide further eco-evolutionary insights at a higher resolution.

## 6. Gut Microbiota in Other Social Insects

There are other eusocial insects whose gut microbial communities have been investigated. Eusocial wasps and hornets, belonging to the family Vespidae in Hymenoptera, have evolved their social lifestyles independently of bees and ants [31]. Those adults feed on plant sources and hunt other insects for larvae in the nest. They show trophallaxis like bees and ants, opening up the path through which gut bacteria are shared among individuals. Previous studies on gut microbiotas of hornet species suggest that their gut microbiota are consistent within species. For example, the Asian giant hornet (*Vespa mandarinia*) and Japanese yellow hornet (*Vespa simillima*) show a high similarity of gut microbiota among individual workers within those species [108]. Their microbiotas are dominated by seven to eight OTUs, three of which are shared by both species, possibly due to the close phylogeny and/or similar ecology of those hosts. The phylogenetic analysis found that those dominant OTUs are close relatives of bacteria detected in potential foods for the hosts, such as *Zymomonas* from fermented plant sap and *Gilliamella* from honey bees, suggesting that hornets’ microbiotas mainly consist of environmentally acquired bacteria [108]. *Vespa velutina*, another Asian hornet currently invading Europe, seems to harbor caste-specific bacterial communities: In a worker and gyne (a female destined to become a queen in the next season), *Gammaproteobacteria* (*Pseudomonadota*) and *Bacilli* (*Bacillota*) highly dominate their gut microbial communities, while workers have less *Lactobacilli*, *Alphaproteobacteria*, and *Actinomycetota*, but more *Buttiauxella* (*Pseudomonadota*) than gynes [109].

Some aphid and thrip species are known to exhibit eusocial lifestyles [110,111,112]. Although microbiomes sampled from whole bodies have been surveyed in some aphids [113,114], gut microbial communities of eusocial aphids and thrips have not been investigated specifically. Aphids are rather used as a model to study mutualistic relationships with obligate symbionts, such as *Buchnera* [115].

## 7. Concluding Remarks and Future Perspectives

Comparing the basic structures of gut bacterial communities of the three eusocial insect groups (eusocial bees, ants, and termites), we could find some patterns of community structures. First, *Pseudomonadota* is the most prevalent taxon ubiquitously associated with those eusocial insects. Secondly, *Bacillota* is commonly detected in eusocial bees and termites but not always in ant species. The presence of those two phyla across host groups might be meaningful, but their compositions at lower taxonomic levels are different. *Snodgrassella* and *Gilliamella* (*Pseudomonoadota*), *Bombilactobacillus* Firm-4, and *Lactobacillus* Firm-5 (*Bacillota*) are specific for eusocial bees but never detected in ant or termite guts. Thirdly, some bacterial genera are highly shared within each group of eusocial bees and termites across host species: *Lactobacillus* (*Bacillota*) and *Bifidobacterium* (*Actinomycetota*) in eusocial bees and *Treponema* (*Spirochaetota*) and *Alistipes* (*Bacteroidota*) in termites. Although their relative abundances in their gut communities vary among host species, they must play crucial roles in symbiosis. Indeed, some of the relevant functions to their presence in those hosts have been reported previously [8,20,106].

Another finding is that ants exhibit significantly distinct gut bacterial communities among host species, compared to eusocial bees and termites. A possible explanation for the huge variation could be the explosive diversification of ant species (eusocial bees, ~860 species; termites, ~3000 species; ants, ~13,000 species). In addition, a more plausible mechanism could be their wide range of dietary habits. Ants include herbivorous, carnivorous, and omnivorous species, while all eusocial bees and termites are herbivores. Intriguingly, some omnivorous and carnivorous ants have few or no gut microbes at the detectable level, while herbivorous species harbor abundantly [79,82,85]. These clearly indicate that host food habit influences the composition of the gut bacterial community. Infection of endosymbiont (e.g., *Wolbachia*) is another important factor generating drastic differences in gut microbiota between infected and uninfected species, as seen in some ants [87].

Eusocial insects tend to share intraspecific gut bacterial communities among individuals within species. This could be promoted by unique food habits and/or social interactions, recurrently suggested by many works of literature [19,50,79,108]. In particular, eusocial insects having narrow dietary habits (e.g., honey bees and termites) are associated with unique gut bacterial species, which have coevolved with hosts. Despite the presence of intraspecific microbial communities, symbiotic relationships between eusocial insects and gut microbes retain some degree of flexibility. Structural differences in gut microbiota become obvious in some eusocial insects when the samples from different castes are compared (Figure 2). It thus seems likely that physiological and ecological changes by social status affect the gut bacterial community. Another representative case is that some carnivorous and omnivorous ant colonies often contain some individuals who do not have detectable gut bacteria, irrespective of their castes. Although the absence of microbes still remains an enigma, some social insects may not rely on a functional gut bacterial community.

Host sociality has a large impact on shaping, maintaining, and specializing gut microbiota, as frequent interactions between individuals result in the long-term association of microbes with the host species. This could serve as a driving force for developing consistent community structures within host species. On the other hand, highly social systems, such as the division of labor or task allocation within a population, diversify the behavior and physiology of individuals, which could induce the alteration of gut microbiota. A fundamental question is, thus, how host insects and gut microbes develop symbiotic relationships with those ecological contexts. Changing the abundance of microbes without altering community compositions might be a promising solution, although further experimental approaches using various eusocial insects are required to test the possibility and unveil its mechanisms.

## Data Availability

All data are provided in the manuscript.

## Supplementary Materials

The following supporting information can be downloaded at https://www.mdpi.com/article/10.3390/insects14050444/s1. Table S1: Meta-information of reference data for Figure 1; Table S2: Meta-information of reference data for Figure 2.

## References

[1] Sommer F., Backhed F. (2013). The gut microbiota-masters of host development and physiology. Nat. Rev. Microbiol..

[2] Moeller A.H., Foerster S., Wilson M.L., Pusey A.E., Hahn B.H., Ochman H. (2016). Social behavior shapes the chimpanzee pan-microbiome. Sci. Adv..

[3] Raulo A., Ruokolainen L., Lane A., Amato K., Knight R., Leigh S., Stumpf R., White B., Nelson K.E., Baden A.L. (2018). Social behaviour and gut microbiota in red-bellied lemurs (*Eulemur rubriventer*): In search of the role of immunity in the evolution of sociality. J. Anim. Ecol..

[4] Lagkouvardos I., Pukall R., Abt B., Foesel B.U., Meier-Kolthoff J.P., Kumar N., Bresciani A., Martinez I., Just S., Ziegler C. (2016). The Mouse Intestinal Bacterial Collection (miBC) provides host-specific insight into cultured diversity and functional potential of the gut microbiota. Nat. Microbiol..

[5] Engel P., Moran N.A. (2013). The gut microbiota of insects—Diversity in structure and function. FEMS Microbiol. Rev..

[6] Bisch G., Neuvonen M.M., Pierce N.E., Russell J.A., Koga R., Sanders J.G., Lukasik P., Andersson S.G.E. (2018). Genome Evolution of Bartonellaceae Symbionts of Ants at the Opposite Ends of the Trophic Scale. Genome Biol. Evol..

[7] Hu Y., Sanders J.G., Lukasik P., D’Amelio C.L., Millar J.S., Vann D.R., Lan Y., Newton J.A., Schotanus M., Kronauer D.J.C. (2018). Herbivorous turtle ants obtain essential nutrients from a conserved nitrogen-recycling gut microbiome. Nat. Commun..

[8] Kesnerova L., Mars R.A.T., Ellegaard K.M., Troilo M., Sauer U., Engel P. (2017). Disentangling metabolic functions of bacteria in the honey bee gut. PLoS Biol..

[9] Hammer T.J., Moran N.A. (2019). Links between metamorphosis and symbiosis in holometabolous insects. Philos. Trans. R Soc. Lond. B Biol. Sci..

[10] Moll R.M., Romoser W.S., Modrzakowski M.C., Moncayo A.C., Lerdthusnee K. (2001). Meconial peritrophic membranes and the fate of midgut bacteria during mosquito (Diptera: Culicidae) metamorphosis. J. Med. Entomol..

[11] Bright M., Bulgheresi S. (2010). A complex journey: Transmission of microbial symbionts. Nat. Rev. Microbiol..

[12] Hosokawa T., Kikuchi Y., Fukatsu T. (2007). How many symbionts are provided by mothers, acquired by offspring, and needed for successful vertical transmission in an obligate insect-bacterium mutualism?. Mol. Ecol..

[13] Hongoh Y., Deevong P., Inoue T., Moriya S., Trakulnaleamsai S., Ohkuma M., Vongkaluang C., Noparatnaraporn N., Kudo T. (2005). Intra- and interspecific comparisons of bacterial diversity and community structure support coevolution of gut microbiota and termite host. Appl. Environ. Microbiol..

[14] Martinson V.G., Moy J., Moran N.A. (2012). Establishment of Characteristic Gut Bacteria during Development of the Honeybee Worker. Appl. Environ. Microbiol..

[15] Powell J.E., Martinson V.G., Urban-Mead K., Moran N.A. (2014). Routes of Acquisition of the Gut Microbiota of the Honey Bee Apis mellifera. Appl. Environ. Microbiol..

[16] Martinson V.G., Danforth B.N., Minckley R.L., Rueppell O., Tingek S., Moran N.A. (2011). A simple and distinctive microbiota associated with honey bees and bumble bees. Mol. Ecol..

[17] Shapira M. (2016). Gut Microbiotas and Host Evolution: Scaling Up Symbiosis. Trends Ecol. Evol..

[18] Koch H., Abrol D.P., Li J., Schmid-Hempel P. (2013). Diversity and evolutionary patterns of bacterial gut associates of corbiculate bees. Mol. Ecol..

[19] Kwong W.K., Moran N.A. (2016). Gut microbial communities of social bees. Nat. Rev. Microbiol..

[20] Brune A. (2014). Symbiotic digestion of lignocellulose in termite guts. Nat. Rev. Microbiol..

[21] Hammer T.J., Le E., Martin A.N., Moran N.A. (2021). The gut microbiota of bumblebees. Insectes Soc..

[22] Hornett E.A., Kageyama D., Hurst G.D.D. (2022). Sex determination systems as the interface between male-killing bacteria and their hosts. Proc. Biol. Sci..

[23] Kaur R., Shropshire J.D., Cross K.L., Leigh B., Mansueto A.J., Stewart V., Bordenstein S.R., Bordenstein S.R. (2021). Living in the endosymbiotic world of Wolbachia: A centennial review. Cell Host. Microbe..

[24] McCutcheon J.P., Boyd B.M., Dale C. (2019). The Life of an Insect Endosymbiont from the Cradle to the Grave. Curr. Biol..

[25] Leonhardt S.D., Menzel F., Nehring V., Schmitt T. (2016). Ecology and Evolution of Communication in Social Insects. Cell.

[26] Holldobler B., Wilson E.O. (1990). The Ants.

[27] Goulson D., Peat J., Stout J.C., Tucker J., Darvill B., Derwent L.C., Hughes W.O.H. (2002). Can alloethism in workers of the bumblebee, Bombus terrestris, be explained in terms of foraging efficiency?. Anim. Behav..

[28] Gruter C., Menezes C., Imperatriz-Fonseca V.L., Ratnieks F.L. (2012). A morphologically specialized soldier caste improves colony defense in a neotropical eusocial bee. Proc. Natl. Acad. Sci. USA.

[29] Koto A., Motoyama N., Tahara H., McGregor S., Moriyama M., Okabe T., Miura M., Keller L. (2019). Oxytocin/vasopressin-like peptide inotocin regulates cuticular hydrocarbon synthesis and water balancing in ants. Proc. Natl. Acad. Sci. USA.

[30] Seid M.A., Traniello J.F.A. (2006). Age-related repertoire expansion and division of labor in Pheidole dentata (Hymenoptera: Formicidae): A new perspective on temporal polyethism and behavioral plasticity in ants. Behav. Ecol. Sociobiol..

[31] Peters R.S., Krogmann L., Mayer C., Donath A., Gunkel S., Meusemann K., Kozlov A., Podsiadlowski L., Petersen M., Lanfear R. (2017). Evolutionary History of the Hymenoptera. Curr. Biol..

[32] Mateus S., Ferreira-Caliman M.J., Menezes C., Grüter C. (2019). Beyond temporal-polyethism: Division of labor in the eusocial bee Melipona marginata. Insectes Sociaux.

[33] Zheng H., Nishida A., Kwong W.K., Koch H., Engel P., Steele M.I., Moran N.A. (2016). Metabolism of Toxic Sugars by Strains of the Bee Gut Symbiont Gilliamella apicola. MBio.

[34] Holtof M., Lenaerts C., Cullen D., Vanden Broeck J. (2019). Extracellular nutrient digestion and absorption in the insect gut. Cell Tissue Res..

[35] Han F., Wallberg A., Webster M.T. (2012). From where did the Western honeybee (*Apis mellifera*) originate?. Ecol. Evol..

[36] Raffiudin R., Crozier R.H. (2007). Phylogenetic analysis of honey bee behavioral evolution. Mol. Phylogenet. Evol..

[37] Winston M.L. (1991). The Biology of the Honey Bee.

[38] Kwong W.K., Medina L.A., Koch H., Sing K.W., Soh E.J.Y., Ascher J.S., Jaffe R., Moran N.A. (2017). Dynamic microbiome evolution in social bees. Sci. Adv..

[39] McFrederick Q.S., Cannone J.J., Gutell R.R., Kellner K., Plowes R.M., Mueller U.G. (2013). Specificity between lactobacilli and hymenopteran hosts is the exception rather than the rule. Appl. Environ. Microbiol..

[40] Engel P., Martinson V.G., Moran N.A. (2012). Functional diversity within the simple gut microbiota of the honey bee. Proc. Natl. Acad. Sci. USA.

[41] Zheng H., Powell J.E., Steele M.I., Dietrich C., Moran N.A. (2017). Honeybee gut microbiota promotes host weight gain via bacterial metabolism and hormonal signaling. Proc. Natl. Acad. Sci. USA.

[42] Emery O., Schmidt K., Engel P. (2017). Immune system stimulation by the gut symbiont Frischella perrara in the honey bee (*Apis mellifera*). Mol. Ecol..

[43] Kwong W.K., Mancenido A.L., Moran N.A. (2017). Immune system stimulation by the native gut microbiota of honey bees. R. Soc. Open Sci..

[44] Kapheim K.M., Rao V.D., Yeoman C.J., Wilson B.A., White B.A., Goldenfeld N., Robinson G.E. (2015). Caste-specific differences in hindgut microbial communities of honey bees (*Apis mellifera*). PLoS ONE.

[45] Ellegaard K.M., Engel P. (2019). Genomic diversity landscape of the honey bee gut microbiota. Nat. Commun..

[46] Jones J.C., Fruciano C., Marchant J., Hildebrand F., Forslund S., Bork P., Engel P., Hughes W.O.H. (2018). The gut microbiome is associated with behavioural task in honey bees. Insectes Soc..

[47] Anderson K.E., Ricigliano V.A., Mott B.M., Copeland D.C., Floyd A.S., Maes P. (2018). The queen’s gut refines with age: Longevity phenotypes in a social insect model. Microbiome.

[48] Boucias D.G., Cai Y., Sun Y., Lietze V.U., Sen R., Raychoudhury R., Scharf M.E. (2013). The hindgut lumen prokaryotic microbiota of the termite Reticulitermes flavipes and its responses to dietary lignocellulose composition. Mol. Ecol..

[49] Figueroa L.L., Maccaro J.J., Krichilsky E., Yanega D., McFrederick Q.S. (2021). Why Did the Bee Eat the Chicken? Symbiont Gain, Loss, and Retention in the Vulture Bee Microbiome. mBio.

[50] Hu Y., Lukasik P., Moreau C.S., Russell J.A. (2014). Correlates of gut community composition across an ant species (*Cephalotes varians*) elucidate causes and consequences of symbiotic variability. Mol. Ecol..

[51] Lukasik P., Newton J.A., Sanders J.G., Hu Y., Moreau C.S., Kronauer D.J.C., O’Donnell S., Koga R., Russell J.A. (2017). The structured diversity of specialized gut symbionts of the New World army ants. Mol. Ecol..

[52] Marynowska M., Goux X., Sillam-Dusses D., Rouland-Lefevre C., Halder R., Wilmes P., Gawron P., Roisin Y., Delfosse P., Calusinska M. (2020). Compositional and functional characterisation of biomass-degrading microbial communities in guts of plant fibre- and soil-feeding higher termites. Microbiome.

[53] Otani S., Mikaelyan A., Nobre T., Hansen L.H., Kone N.A., Sorensen S.J., Aanen D.K., Boomsma J.J., Brune A., Poulsen M. (2014). Identifying the core microbial community in the gut of fungus-growing termites. Mol. Ecol..

[54] Sapountzis P., Zhukova M., Hansen L.H., Sorensen S.J., Schiott M., Boomsma J.J. (2015). Acromyrmex Leaf-Cutting Ants Have Simple Gut Microbiota with Nitrogen-Fixing Potential. Appl. Environ. Microbiol..

[55] Segers F., Kaltenpoth M., Foitzik S. (2019). Abdominal microbial communities in ants depend on colony membership rather than caste and are linked to colony productivity. Ecol. Evol..

[56] Ellegaard K.M., Suenami S., Miyazaki R., Engel P. (2020). Vast Differences in Strain-Level Diversity in the Gut Microbiota of Two Closely Related Honey Bee Species. Curr. Biol..

[57] Moran N.A., Hansen A.K., Powell J.E., Sabree Z.L. (2012). Distinctive gut microbiota of honey bees assessed using deep sampling from individual worker bees. PLoS ONE.

[58] Kesnerova L., Emery O., Troilo M., Liberti J., Erkosar B., Engel P. (2020). Gut microbiota structure differs between honeybees in winter and summer. ISME J..

[59] Otani S., Zhukova M., Kone N.A., da Costa R.R., Mikaelyan A., Sapountzis P., Poulsen M. (2019). Gut microbial compositions mirror caste-specific diets in a major lineage of social insects. Environ. Microbiol. Rep..

[60] Belsky J.E., Camp A.A., Lehmann D.M. (2020). The Importance of Males to Bumble Bee (Bombus Species) Nest Development and Colony Viability. Insects.

[61] Sadd B.M., Barribeau S.M., Bloch G., de Graaf D.C., Dearden P., Elsik C.G., Gadau J., Grimmelikhuijzen C.J., Hasselmann M., Lozier J.D. (2015). The genomes of two key bumblebee species with primitive eusocial organization. Genome Biol..

[62] Powell E., Ratnayeke N., Moran N.A. (2016). Strain diversity and host specificity in a specialized gut symbiont of honeybees and bumblebees. Mol. Ecol..

[63] Martinson V.G., Magoc T., Koch H., Salzberg S.L., Moran N.A. (2014). Genomic features of a bumble bee symbiont reflect its host environment. Appl. Environ. Microbiol..

[64] Killer J., Kopecny J., Mrazek J., Havlik J., Koppova I., Benada O., Rada V., Kofronova O. (2010). *Bombiscardovia coagulans* gen. nov., sp. nov., a new member of the family Bifidobacteriaceae isolated from the digestive tract of bumblebees. Syst. Appl. Microbiol..

[65] Koch H., Schmid-Hempel P. (2011). Socially transmitted gut microbiota protect bumble bees against an intestinal parasite. Proc. Natl. Acad. Sci. USA.

[66] Leger L., McFrederick Q.S. (2020). The Gut-Brain-Microbiome Axis in Bumble Bees. Insects.

[67] Li L., Solvi C., Zhang F., Qi Z., Chittka L., Zhao W. (2021). Gut microbiome drives individual memory variation in bumblebees. Nat. Commun..

[68] Li J., Powell J.E., Guo J., Evans J.D., Wu J., Williams P., Lin Q., Moran N.A., Zhang Z. (2015). Two gut community enterotypes recur in diverse bumblebee species. Curr. Biol..

[69] Parmentier A., Meeus I., Van Nieuwerburgh F., Deforce D., Vandamme P., Smagghe G. (2018). A different gut microbial community between larvae and adults of a wild bumblebee nest (*Bombus pascuorum*). Insect Sci..

[70] Wang L., Wu J., Li K., Sadd B.M., Guo Y., Zhuang D., Zhang Z., Chen Y., Evans J.D., Guo J. (2019). Dynamic Changes of Gut Microbial Communities of Bumble Bee Queens through Important Life Stages. mSystems.

[71] Bosmans L., Pozo M.I., Verreth C., Crauwels S., Wackers F., Jacquemyn H., Lievens B. (2018). Hibernation Leads to Altered Gut Communities in Bumblebee Queens (Bombus terrestris). Insects.

[72] Rasmussen C., Cameron S.A. (2009). Global stingless bee phylogeny supports ancient divergence, vicariance, and long distance dispersal. Biol. J. Linn. Soc..

[73] Hartfelder K., Makert G.R., Judice C.C., Pereira G.A.G., Santana W.C., Dallacqua R., Bitondi M.M.G. (2006). Physiological and genetic mechanisms underlying caste development, reproduction and division of labor in stingless bees. Apidologie.

[74] Gruter C., Segers F.H., Menezes C., Vollet-Neto A., Falcon T., von Zuben L., Bitondi M.M., Nascimento F.S., Almeida E.A. (2017). Repeated evolution of soldier sub-castes suggests parasitism drives social complexity in stingless bees. Nat. Commun..

[75] Sarton-Loheac G., Nunes da Silva C.G., Mazel F., Baud G., de Bakker V., Das S., El Chazli Y., Ellegaard K., Garcia-Garcera M., Glover N. (2023). Deep Divergence and Genomic Diversification of Gut Symbionts of Neotropical Stingless Bees. mBio.

[76] Cerqueira A.E.S., Hammer T.J., Moran N.A., Santana W.C., Kasuya M.C.M., da Silva C.C. (2021). Extinction of anciently associated gut bacterial symbionts in a clade of stingless bees. ISME J..

[77] Roubik D.W. (1982). Obligate necrophagy in a social bee. Science.

[78] Hall M.A., Brettell L.E., Liu H., Nacko S., Spooner-Hart R., Riegler M., Cook J.M. (2020). Temporal changes in the microbiome of stingless bee foragers following colony relocation. FEMS Microbiol. Ecol..

[79] Moreau C.S. (2020). Symbioses among ants and microbes. Curr. Opin. Insect Sci..

[80] Cook S.C., Davidson D.W. (2006). Nutritional and functional biology of exudate-feeding ants. Entomologia Experimentalis Applicata.

[81] Roche R.K., Wheeler D.E. (1997). Morphological specializations of the digestive tract ofZacryptocerus rohweri (Hymenoptera: Formicidae). J. Morphol..

[82] Sanders J.G., Lukasik P., Frederickson M.E., Russell J.A., Koga R., Knight R., Pierce N.E. (2017). Dramatic Differences in Gut Bacterial Densities Correlate with Diet and Habitat in Rainforest Ants. Integr. Comp. Biol..

[83] Anderson K.E., Russell J.A., Moreau C.S., Kautz S., Sullam K.E., Hu Y., Basinger U., Mott B.M., Buck N., Wheeler D.E. (2012). Highly similar microbial communities are shared among related and trophically similar ant species. Mol. Ecol..

[84] Urbani C.B., de Andrade M.L. (1997). Pollen Eating, Storing, and Spitting by Ants. Naturwissenschaften.

[85] Russell J.A., Moreau C.S., Goldman-Huertas B., Fujiwara M., Lohman D.J., Pierce N.E. (2009). Bacterial gut symbionts are tightly linked with the evolution of herbivory in ants. Proc. Natl. Acad. Sci. USA.

[86] Andersen S.B., Hansen L.H., Sapountzis P., Sorensen S.J., Boomsma J.J. (2013). Specificity and stability of the Acromyrmex-Pseudonocardia symbiosis. Mol. Ecol..

[87] Zhukova M., Sapountzis P., Schiott M., Boomsma J.J. (2017). Diversity and Transmission of Gut Bacteria in Atta and Acromyrmex Leaf-Cutting Ants during Development. Front. Microbiol..

[88] Hammer T.J., Sanders J.G., Fierer N. (2019). Not all animals need a microbiome. FEMS Microbiol. Lett..

[89] Russell J.A., Sanders J.G., Moreau C.S. (2017). Hotspots for symbiosis: Function, evolution, and specificity of ant-microbe associations from trunk to tips of the ant phylogeny (Hymenoptera: Formicidae). Myrmecol. News..

[90] Brown B.P., Wernegreen J.J. (2016). Deep divergence and rapid evolutionary rates in gut-associated Acetobacteraceae of ants. BMC Microbiol..

[91] Koto A., Nobu M.K., Miyazaki R. (2020). Deep Sequencing Uncovers Caste-Associated Diversity of Symbionts in the Social Ant Camponotus japonicus. mBio.

[92] Moreau C.S., Rubin B.E.R. (2017). Diversity and Persistence of the Gut Microbiome of the Giant Neotropical Bullet Ant. Integr. Comp. Biol..

[93] Chouvenc T., Sobotnik J., Engel M.S., Bourguignon T. (2021). Termite evolution: Mutualistic associations, key innovations, and the rise of Termitidae. Cell Mol. Life Sci..

[94] Bucek A., Sobotnik J., He S., Shi M., McMahon D.P., Holmes E.C., Roisin Y., Lo N., Bourguignon T. (2019). Evolution of Termite Symbiosis Informed by Transcriptome-Based Phylogenies. Curr. Biol..

[95] Mikaelyan A., Dietrich C., Kohler T., Poulsen M., Sillam-Dusses D., Brune A. (2015). Diet is the primary determinant of bacterial community structure in the guts of higher termites. Mol. Ecol..

[96] Mikaelyan A., Meuser K., Brune A. (2017). Microenvironmental heterogeneity of gut compartments drives bacterial community structure in wood- and humus-feeding higher termites. FEMS Microbiol. Ecol..

[97] Donovan S.E., Jones D.T., Sands W.A., Eggleton P. (2000). Morphological phylogenetics of termites (Isoptera). Biol. J. Linn. Soc..

[98] Ohkuma M. (2008). Symbioses of flagellates and prokaryotes in the gut of lower termites. Trends Microbiol..

[99] Berlanga M., Paster B.J., Grandcolas P., Guerrero R. (2011). Comparison of the gut microbiota from soldier and worker castes of the termite Reticulitermes grassei. Int. Microbiol..

[100] Cameron S.L., Lo N., Bourguignon T., Svenson G.J., Evans T.A. (2012). A mitochondrial genome phylogeny of termites (Blattodea: Termitoidae): Robust support for interfamilial relationships and molecular synapomorphies define major clades. Mol. Phylogenet. Evol..

[101] Reid N.M., Addison S.L., West M.A., Lloyd-Jones G. (2014). The bacterial microbiota of Stolotermes ruficeps (Stolotermitidae), a phylogenetically basal termite endemic to New Zealand. FEMS Microbiol. Ecol..

[102] Dietrich C., Kohler T., Brune A. (2014). The cockroach origin of the termite gut microbiota: Patterns in bacterial community structure reflect major evolutionary events. Appl. Environ. Microbiol..

[103] Hyodo F., Tayasu I., Inoue T., Azuma J.I., Kudo T., Abe T. (2003). Differential role of symbiotic fungi in lignin degradation and food provision for fungus-growing termites (Macrotermitinae: Isoptera). Funct. Ecol..

[104] Su L., Yang L., Huang S., Su X., Li Y., Wang F., Wang E., Kang N., Xu J., Song A. (2016). Comparative Gut Microbiomes of Four Species Representing the Higher and the Lower Termites. J. Insect Sci..

[105] Mikaelyan A., Kohler T., Lampert N., Rohland J., Boga H., Meuser K., Brune A. (2015). Classifying the bacterial gut microbiota of termites and cockroaches: A curated phylogenetic reference database (DictDb). Syst. Appl. Microbiol..

[106] Tokuda G., Mikaelyan A., Fukui C., Matsuura Y., Watanabe H., Fujishima M., Brune A. (2018). Fiber-associated spirochetes are major agents of hemicellulose degradation in the hindgut of wood-feeding higher termites. Proc. Natl. Acad. Sci. USA.

[107] Bourguignon T., Lo N., Dietrich C., Sobotnik J., Sidek S., Roisin Y., Brune A., Evans T.A. (2018). Rampant Host Switching Shaped the Termite Gut Microbiome. Curr. Biol..

[108] Suenami S., Konishi Nobu M., Miyazaki R. (2019). Community analysis of gut microbiota in hornets, the largest eusocial wasps, Vespa mandarinia and V. simillima. Sci. Rep..

[109] Cini A., Meriggi N., Bacci G., Cappa F., Vitali F., Cavalieri D., Cervo R. (2020). Gut microbial composition in different castes and developmental stages of the invasive hornet Vespa velutina nigrithorax. Sci. Total Environ..

[110] Crespi B.J. (1992). Eusociality in Australian gall thrips. Nature.

[111] Kranz B.D., Schwarz M.P., Mound L.A., Crespi B.J. (1999). Social biology and sex ratios of the eusocial gall-inducing thripsKladothrips hamiltoni. Ecol. Entomol..

[112] Shibao H., Kutsukake M., Fukatsu T. (2021). Temporal division of labor in an aphid social system. Sci. Rep..

[113] Xu T.T., Chen J., Jiang L.Y., Qiao G.X. (2021). Diversity of bacteria associated with Hormaphidinae aphids (Hemiptera: Aphididae). Insect Sci..

[114] Xu T.T., Jiang L.Y., Chen J., Qiao G.X. (2020). Host Plants Influence the Symbiont Diversity of Eriosomatinae (Hemiptera: Aphididae). Insects.

[115] Shigenobu S., Yorimoto S. (2022). Aphid hologenomics: Current status and future challenges. Curr. Opin. Insect Sci..

